# The Activated Macrophage – A Tough Fortress for Virus Invasion: How Viruses Strike Back

**DOI:** 10.3389/fmicb.2021.803427

**Published:** 2022-01-11

**Authors:** Andra Banete, Julia Barilo, Reese Whittaker, Sameh Basta

**Affiliations:** ^1^Department of Biomedical and Molecular Sciences, Queen’s University, Kingston, ON, Canada; ^2^Department of Biological Sciences, Sunnybrook Research Institute, University of Toronto, Toronto, ON, Canada

**Keywords:** cytokines, polarized macrophages, interleukin-4, interferon-gamma, virus

## Abstract

Macrophages (Mφ) are innate immune cells with a variety of functional phenotypes depending on the cytokine microenvironment they reside in. Mφ exhibit distinct activation patterns that are found within a wide array of activation states ranging from the originally discovered classical pro-inflammatory (M1) to the anti-inflammatory (M2) with their multi-facades. M1 cells are induced by IFNγ + LPS, while M2 are further subdivided into M2a (IL-4), M2b (Immune Complex) and M2c (IL-10) based on their inducing stimuli. Not surprisingly, Mφ activation influences the outcome of viral infections as they produce cytokines that in turn activate cells of the adaptive immune system. Generally, activated M1 cells tend to restrict viral replication, however, influenza and HIV exploit inflammation to support their replication. Moreover, M2a polarization inhibits HIV replication at the post-integration level, while HCMV encoded hrIL-10 suppresses inflammatory reactions by facilitating M2c formation. Additionally, viruses such as LCMV and Lassa Virus directly suppress Mφ activation leading to viral chronicity. Here we review how Mφ activation affects viral infection and the strategies by which viruses manipulate Mφ polarization to benefit their own fitness. An understanding of these mechanisms is important for the development of novel immunotherapies that can sway Mφ phenotype to inhibit viral replication.

## Activated Macrophages: The Foe to Viral Infections

Macrophages (Mφ) recognize viruses, and bridge innate and adaptive immunity to assist in T cell priming (Naito et al., 1996; Takahashi et al., 1996; Iwasaki and Medzhitov, 2010; Trus et al., 2020; Petrina et al., 2021). Mφ polarization (Murray et al., 2014; Yunna et al., 2020), occurs in response to changing environmental stimuli where activated Mφ can become (M1) associated with Th1 cytokines response, or (M2) associated with Th2 cytokines (Nathan et al., 1983; Stein et al., 1992; Gordon and Taylor, 2005; Yunna et al., 2020). The pro-inflammatory M1 phenotype is induced by lipopolysaccharide (LPS) in the presence of interferon-gamma (IFNγ) (Nathan et al., 1983; Nathan and Hibbs, 1991; Tugal et al., 2013). In contrast, M2 Mφ function as anti-inflammatory cells and promote tissue repair (Stein et al., 1992; Martinez et al., 2006; Tugal et al., 2013). The M2 designation has been further subdivided into (M2a, b, c, and d) based on the cytokines that induces them and their gene expression profiles (Mantovani et al., 2004). For example, M2a Mφ, involved in parasitic infections (Sica and Mantovani, 2012), can be derived from either bone marrow or spleen tissues by short or long-term incubation with IL-4 (Mulder et al., 2017; Banete et al., 2021). Genetic approaches have been used to investigate Mφ activation (Biswas and Mantovani, 2010; Smale, 2010; Lawrence and Natoli, 2011), but little is known about dysregulated Mφ functions during viral infection (Beadling and Slifka, 2004; Trivedi et al., 2018).

In various infectious diseases, activated Mφ produce cytokines such as IL-6, IL-12 and IL-23 to regulate immunity against viral invasion (Arango Duque and Descoteaux, 2014; Ruytinx et al., 2018). IL-12 and IL-23 are induced by diverse Mφ populations after Toll-like receptor (TLR) stimulation by viral pathogen-associated molecular patterns (PAMPs) (Gee et al., 2009; Mehta et al., 2015; Petes et al., 2017; Che Mat et al., 2018; Alothaimeen et al., 2021; Banete et al., 2021).

When Mφ encounter viruses or viral PAMPs, they begin producing type I IFNs, which are needed to aid with NK and eventually T cell activation (Kadowaki and Liu, 2002; Keppler et al., 2012). This IFN rapid response starts with the production of IFN-β followed by IFN-α, causing the phosphorylation of interferon regulatory factor 7 (IRF7), which is needed to enhance the antiviral response (Sun et al., 2013). IRFs can also be activated *via* the endoplasmic reticulum (ER) adaptor protein stimulator of interferon genes (STING), which detects cytoplasmic DNA from viral infection, resulting in type I IFN induction that interferes with virus replication (Banete et al., 2018). Several viruses have been shown to interfere with this STING-induced type I IFN response (Banete et al., 2018). For example, Dengue virus (DENV) expresses a protease (NS2B3) that cleaves STING causing the reduction of induced type I IFN after infection with DENV (Aguirre et al., 2012).

Other viruses, such as Herpes simplex virus (HSV), have been shown to interfere with type I IFN production in human Mφ after infection by expressing a viral inhibitory protein that interferes with the STING signalosome activation of IRF (Christensen et al., 2016). There is evidence that the TRIM family of proteins are critical in the activation of STING. Recent reports show that TRIM29 is expressed in alveolar Mφ, where they regulate their activation state, acting as a negative regulator of antiviral immune responses (Xing et al., 2017, 2018; Li et al., 2018). TRIM29 was shown to be up-regulated by viral RNA and DNA, with EBV suppressing innate immune responses by targeting STING through the TRIM29 signaling pathway, indicating a mechanism of persistence for DNA viruses. Whether viruses can regulate Mφ polarization through TRIM29 remains to be determined. Type II IFN (IFN-γ) can directly inhibit Murine Norovirus replication by reducing the levels of both structural and non-structural viral proteins expression in infected cells (Changotra et al., 2009). Additionally, the ability of IFN-γ to induce nitric oxide synthase (iNOS) to aid in how Mφ respond to viruses to inhibit Coxsackievirus viral replication (Jarasch et al., 2005). Viruses have developed mechanisms to evade this activation pathway; for example Epstein-Barr virus (EBV) encodes several proteins that inhibit IFN-γ as well as type I IFNs by targeting the activation of the JAK-STAT signaling pathways (Taylor et al., 2015; Jangra et al., 2021).

In addition to IFNs, other cytokines secreted by activated Mφ, such as IL-1β, and IL-6, can also contribute to antiviral activities due to their ability to activate MAPK/ERK signaling pathways (Lucin et al., 1994; Ichikawa et al., 2002; O’Neill, 2008; Tanaka et al., 2014; Mariani et al., 2019). Thus, activated Mφ can help mediate viral restriction by producing a variety of cytokines which could also be influenced by their polarization states.

Studies on HIV-1 have characterized some of the antiviral responses observed in Mφ (Cassol et al., 2009; Li et al., 2009). In HIV-1, M2a Mφ inhibit virus replication to a certain degree without impairing viral entry or reverse transcriptase activities, suggesting that inhibition occurs in the later events of the viral cycle, while M1 cells downregulate CD4 expression to prevent HIV-1 entry into cells (Cassol et al., 2009). The mechanism behind the impaired virus replication in M2a cells has not been fully elucidated in the above model. Though, in other viral infections, HSV-1 replication was observed to be significantly higher in M2 than M1 cells *in vitro*, but *in vivo*, M2 cells were better at restricting viral replication (Lee and Ghiasi, 2017). Thus, viral inhibition by M2a cells could be limited to certain viral models. Indeed, more studies are needed to carefully delineate the mechanism of antiviral responses in activated Mφ, because certain virus infections could benefit from the Mφ polarization status.

## How Viruses Counter Mφ Activation

Mφ are early targets for viral infection, and their activation by infection plays a crucial role in regulating innate and adaptive immunity. Many studies show that pathogenic Arenaviruses such as LASV and Junin virus (JUNV) are highly immunosuppressive, in contrast with their non-pathogenic counterparts Mopeia virus (MOPV) and Tacaribe virus (TCRV). Infection does not activate human Mφ upon infection, and patients who succumb to hemorrhagic disease lack a significant upregulation of pro-inflammatory cytokines in their sera (Baize et al., 2004). As well, the pathogenic LCMV has also been shown to inhibit Mφ activation, in contrast to Pichinde arenavirus, which does not cause disease in humans (Xing et al., 2015a). LCMV infection of human monocyte-derived Mφ does not up-regulate cytokine production and the co-stimulatory molecules CD80 and CD86, leading to the inhibition of Mφ activation (Xing et al., 2015a).

Furthermore, infection with human cytomegalovirus (HCMV) is associated with immunological dysfunction. Studies show that HCMV encodes different gene products that can modulate immune functions to enhance viral pathogenesis. *UL111A* encodes homologs of the anti-inflammatory cytokine human IL-10 during both the acute and latent stages of infection (Kotenko et al., 2000; Jenkins et al., 2004). Mφ polarization is skewed toward a deactivated M2c phenotype, with downregulated pro-inflammatory cytokine production, and inhibition of MHC I and II expression (Avdic et al., 2013). As well, M2c polarization by HCMV viral IL-10 reduces their ability to stimulate CD4 T cell activation and proliferation (Avdic et al., 2013). Thus, it is crucial to further understand how viruses can manipulate the activation state of Mφ to benefit their own replication, as it may help in the development of immunotherapies.

As highlighted above, M1 Mφ are associated with the production of inflammatory cytokines (Sica et al., 2015), and are prominent in the initial stages of the antiviral immune response (Gracia-Hernandez et al., 2020). Thus, Mφ represent key targets for viruses to infect (Sang et al., 2015). This has been observed in viral infections such as with HIV, where Mφ act as a reservoir for HIV due to their long half-life. African Swine Fever Virus (ASFV) is another virus that infects many types of Mφ (Basta et al., 1999, 2001; McCullough et al., 1999). *In vivo*, increased numbers of Mφ after ASFV infections were observed and they tend play a role in ASF viral pathogenesis by contributing to increased levels of proinflammatory cytokines, typically associated with M1 cells that can cause severe pathology (Gomez-Villamandos et al., 2013; Paulina Achita, 2015).

The role IL-6 plays during viral clearance has been shown to favor viral infections in mice infected with Theiler’s murine encephalomyelitis virus (Hou et al., 2014). In this study, excessive levels of the IL-6 cytokine were observed due to viral infection. This causes an increased number of inflammatory IL-17-producing helper T cells. The combined effects of IL-6 and IL-17 synergistically allow for viral persistence because virus-infected cells were protected from undergoing apoptosis (Hou et al., 2014). Thus, high levels of IL-6 benefit the virus in this infection model.

The excessive polarization of M1 and M2 Mφ can be correlated with viral infection and its related complications, such as sepsis and acute respiratory distress syndrome (ARDS) (Zhang et al., 2019). M1-polarized cells can be problematic in certain viral infections, as they recruit other cell populations to the inflammation site, which creates a favorable environment for virus infection of immune cells (Herbein and Varin, 2010; Nikitina et al., 2018). An example of M1 Mφ promoting viral dissemination is in the acute phase of HIV infection. This phase is characterized by a predominance of M1 Mφ expressing the proinflammatory cytokines TNF-α, IL-1β, IL-6 and IL-18 (Burdo et al., 2015; Nikitina et al., 2018). This seems to aid in HIV spread, since inflammation promotes the recruitment of more monocytes and T-cells to the site, which allows HIV to infect these cells and establish infection in the host.

In SARS-CoV-2 severe infections, monocytes are recruited to the lungs where they differentiate into Mφ that tend to upregulate pro-inflammatory genes needed for T-cells activation (Gracia-Hernandez et al., 2020; Zheng et al., 2021). The continuous recruitment of inflammatory monocytes to lung tissues can be harmful to the host as it distorts pulmonary Mφ to persist in the active M1 state (Morales-Nebreda et al., 2015). This bias toward M1 Mφ during viral infection can cause undesirable pathological inflammatory response leading to ARDS (Brufsky, 2020). In ARDS, high levels of inflammatory cytokines especially IL-6 are problematic (Zheng et al., 2021). In this scenario, high levels of pro-inflammatory cytokines enhance viral persistence, multiorgan failure, vascular permeability and possibly death (Gracia-Hernandez et al., 2020).

It has recently been shown that infection of Mφ by SARS-CoV-2 triggers M2-associated gene expression *in vivo*. In addition, infection of polarized M1 and M2 Mφ significantly increases the release of both pro- and anti-inflammatory cytokines after 24 and 48 h. Interestingly, although all Mφ subtypes were susceptible to SARS-CoV-2 infection, viral load was significantly lower in M2 compared to M0 (Boumaza et al., 2021). The higher permissivity of M0 and M1 macrophages to SARS-CoV-2 infection may be why conditions associated with excessive M1 polarization, such as obesity and diabetes, are comorbidities of COVID-19.

Another example where the virus makes use of Mφ is seen in Human Cytomegalovirus (HCMV) where the virus is able to establish a low-level productive infection in both types of Mφ (M1 and M2) for a total of 21 days *in vitro* culture (Bayer et al., 2013). Both types of Mφ display features of activation with upregulation of inflammatory cytokines such as IL-6 and TNF-α (Bayer et al., 2013). It has been shown that HCMV can regulate the polarization of infected Mφ to create a favorable environment for the virus to disseminate (Nikitina et al., 2018). These examples remind us of the importance of studying Mφ responses in different models of viral infections.

## Viral Tricks to Avoid Pathogen Recognition Receptors Activation

Viruses PAMPs are recognized by pathogen recognition receptors (PRRs) expressed by Mφ (Kawai and Akira, 2009). PRRs include Toll-like receptors (TLRs), the retinoic-acid-inducible gene I (RIG-I)-like receptors (RLRs), melanoma differentiation-associated gene 5 (MDA5), and NOD-like receptors (NLRs) (Takeuchi and Akira, 2010). Endosomal TLRs recognize viral nucleic acids. Upon encountering infections, these receptors help in the initiation of immune responses, inducing the influx of inflammatory cells to the site of infection (Biswas and Mantovani, 2010).

**FIGURE 1:**
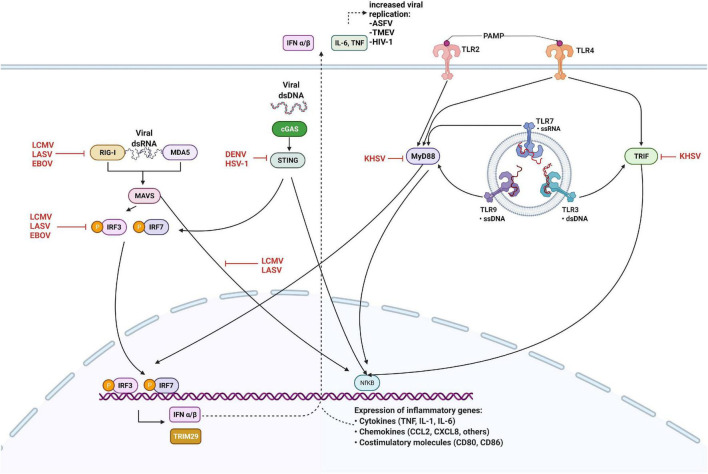
Innate immune antagonism by viruses. Retinoic acid-inducible gene I (RIG-I) and melanoma differentiation-associated gene 5 (MDA5) detect cytosolic viral RNA and interact with MAVS, cGAS binds DNA to activate the STING pathway. Viral PAMPs interact with Toll-like receptors expressed on the plasma membrane and in the endosome, leading to the activation of MyD88 and TRIF. Detection of viral nucleic acid triggers downstream signaling culminate in the phosphorylation and activation of interferon regulatory factors (IRFs) 3 and 7, and expression of type I interferons (IFN) together with NF-kB. Viral proteins inhibit innate sensing and signaling by interacting with cellular proteins at several steps, as shown. Black arrows indicate pathways that lead to the activation of downstream molecules, while red blunted arrows indicate steps that are inhibited. Figure created with licensed Biorender software.

In response to PRRs stimulation (Figure 1), IRFs and NF-κB are activated to induce IFN production and pro-inflammatory cytokines. IRF1, 3, and 7 have been implicated as positive regulators of type I IFN transcription. IRF3 and 7 are essential for the cytosolic pathway induction of type I IFN, whereas IRF1 is non-essential. IFN signaling then initiates a positive feedback loop, acting in autocrine and paracrine manners to induce interferon-stimulated genes (ISGs). Two essential ISGs involved in RNA virus infections are RIG-I and MDA5, recognizing cytoplasmic ssRNA and dsRNA, respectively (Reikine et al., 2014). Upon interaction with their ligands, RIG-I and MDA5 oligomerize to form filaments, interacting with their adaptor protein MAVS to induce filament formation and signaling. Activated MAVS form large, prion-like aggregates (Hou et al., 2011). It has been shown recently that membrane-bound organelles are platforms for immune signaling events (Vazquez and Horner, 2015). In addition to the mitochondria, MAVS has also been found on peroxisomes, where it can induce a unique signaling pathway that specifically triggers IFNλ expression but not IFNβ in response to certain viral infections (Dixit et al., 2010; Odendall et al., 2014; Bender et al., 2015).

Another sensor of RNA virus infection in Mφ is poly ADP-ribose polymerase 9 (PARP9) (Xing et al., 2021). Interestingly, PARP9 is required to control RNA virus infection in STAT1-dependent signal transduction, enhancing IFN-regulated host responses (Zhang et al., 2015). While PARP9 increases M1-associated gene expression (Iwata et al., 2016), PARP14 has been shown to enhance IL-4-dependent gene expression (Mehrotra et al., 2011). All members of the Coronaviridae family encode a macrodomain that reverse ADP-ribosylation by PARP proteins (Grunewald et al., 2019), and recently the SARS-CoV-2 Nsp macrodomain has been shown to impair IFN signaling and induction of IFN-responsive genes (Russo et al., 2021).

Moreover, TLR signaling pathway molecules are targets for viral inhibition. Arenaviruses are able to inhibit a TLR2 response through the suppression of NF-κB activation by the viral NP (Rodrigo et al., 2012). The Arenavirus NP plays several roles in the suppression of immune responses (Martinez-Sobrido et al., 2007). It is able to prevent the nuclear translocation and transcriptional activity of NF-κB by binding the IkB kinase (IKK)-related kinase IKKε, which is part of the upstream complex involved in activation and subsequent translocation of NF-κB to the nucleus (Pythoud et al., 2012). Also, infection of monocytes with Kaposi’s sarcoma-associated herpesvirus (KHSV) inhibits both TLR2 and TLR4 signaling (Lagos et al., 2008; Meyer et al., 2013). The KHSV replication and transcription activator (RTA) induces the degradation of mRNA encoding MyD88. RTA also promotes proteasomal degradation of TLR3 adaptor protein TRIF, which blocks downstream signaling (Bussey et al., 2014).

Furthermore, Arenavirus NP is involved in the suppression of type I IFN responses through an early interference with the IRF3 activation pathway (Martinez-Sobrido et al., 2007). This leads to inhibition of type I IFN production and ISGs expression, needed for the establishment of an antiviral state. IRF3 is normally present in the cytoplasm in an inactive state. However, in response to viral infection, it is phosphorylated and can either dimerize or form a complex with IRF7 to translocate to the nucleus where it activates the transcription of IFNα and IFNβ. LCMV-NP inhibits IRF3 phosphorylation through the same mechanism as NF-κB inhibition (Pythoud et al., 2012). The classical IKK complex IKKα/IKKβ is involved in activation of NF-κB, whereas TBK1 (TANK-binding kinase) and IKKε can also activate IRF by direct phosphorylation of IRF3 and IRF7. By binding to IKKε and inhibiting its function, LCMV-NP inhibits phosphorylation of IRF3, preventing its activation and nuclear translocation (Pythoud et al., 2012). Another viral inhibitory protein, KHSV ORF45 acts as a competitive substrate for IKKε and TBK1 to prevent the activation of IRF7 (Liang et al., 2012). As well, the Ebola virus (EBOV) VP35 blocks TBK1 and IKKε-mediated IRF7 phosphorylation, leading to the inhibition of type I IFN production (Leung et al., 2009; Kimberlin et al., 2010).

Viruses can induce type I IFN production through cytosolic PRRs such as RIG-I and MDA5, which detect viral 5′-triphosphorylated ssRNA and dsRNA present in the cytoplasm of infected cells. When activated by viral dsRNA, RIG-I and MDA5 lead to the activation of signaling pathways that activate IRFs, and NF-κB, that translocate to the nucleus and activate the transcription of inflammatory cytokines and type I IFNs (Figure 1). Arenavirus-NP evades immune detection by both RIG-I and MDA5, because the C-terminal domain of NP has exonuclease activity and can digest dsRNA, preventing its sensing by RIG-I and MDA5 and subsequent production of type I IFNs (Borrow et al., 2010; Hastie et al., 2011; Reynard et al., 2014; Huang et al., 2015). However, this inhibitory mechanism is not fully efficient and small amounts of type I IFN can still be produced.

Arenavirus Z protein is a small zinc-binding protein involved in the regulation of replication and transcription of the virus genome, as well as in the mediation of viral budding (Salvato et al., 1992; Perez et al., 2003; Kranzusch and Whelan, 2011). Introducing the Z protein of pathogenic arenaviruses into non-pathogenic species was shown to enhance viral replication in Mφ, which are the early target of these viruses. Interestingly, it has been reported that the Z protein of all arenaviruses pathogenic to humans, including LCMV, is able to inhibit IFN production by binding to RLRs. The Z protein binds to the N-terminal CARD-domain of RIG-I and MDA5, which disrupts their interaction with MAVS to inhibit downstream signaling (Xing et al., 2015b).

Another well-characterized viral immune inhibitory protein is the EBOV VP35 protein. VP35 prevents the activation of RIG-I signaling by shielding the viral dsRNA from detection by effectively coating the viral genome and preventing its interaction with cytosolic detectors (Leung et al., 2011).

In conclusion, viruses evolved multiple tactics to cope with the host immune response, which taught us many lessons in Microbiology. Activated Mφ antiviral functions can be dictated by their polarization and activation signals they exchange. In certain infections, polarization toward one end of the spectrum may be associated with immunopathology. This imbalance can provide an advantage for viral replication. Lessons gathered from the above studies necessitate more research in understanding and utilizing polarized Mφ in antiviral immunotherapeutics.

## Author Contributions

AB, JB, RW, and SB conceptualized and wrote the review. All authors contributed to the article and approved the submitted version.

## Conflict of Interest

The authors declare that the research was conducted in the absence of any commercial or financial relationships that could be construed as a potential conflict of interest.

## Publisher’s Note

All claims expressed in this article are solely those of the authors and do not necessarily represent those of their affiliated organizations, or those of the publisher, the editors and the reviewers. Any product that may be evaluated in this article, or claim that may be made by its manufacturer, is not guaranteed or endorsed by the publisher.
